# Tumor‐Suppressor p53TAD^1–60^ Forms a Fuzzy Complex with Metastasis‐Associated S100A4: Structural Insights and Dynamics by an NMR/MD Approach

**DOI:** 10.1002/cbic.202000348

**Published:** 2020-07-22

**Authors:** Erika F. Dudás, Gyula Pálfy, Dóra K. Menyhárd, Fanni Sebák, Péter Ecsédi, László Nyitray, Andrea Bodor

**Affiliations:** ^1^ Laboratory of Structural Chemistry and Biology Eötvös Loránd University Pázmány Péter sétány 1/a Budapest 1117 Hungary; ^2^ MTA-ELTE Protein Modelling Research Group Pázmány Péter sétány. 1/a Budapest 1117 Hungary; ^3^ Doctoral School of Pharmaceutical Sciences Semmelweis University Üllői út 26 Budapest 1085 Hungary; ^4^ Department of Biochemistry Eötvös Loránd University Pázmány Péter sétány 1/c Budapest 1117 Hungary

**Keywords:** fuzzy complexes, molecular dynamics simulations, NMR structures, p53, protein folding, S100A4

## Abstract

Conformationally flexible protein complexes represent a major challenge for structural and dynamical studies. We present herein a method based on a hybrid NMR/MD approach to characterize the complex formed between the disordered p53TAD^1–60^ and the metastasis‐associated S100A4. Disorder‐to‐order transitions of both TAD1 and TAD2 subdomains upon interaction is detected. Still, p53TAD^1–60^ remains highly flexible in the bound form, with residues L26, M40, and W53 being anchored to identical hydrophobic pockets of the S100A4 monomer chains. In the resulting “fuzzy” complex, the clamp‐like binding of p53TAD^1–60^ relies on specific hydrophobic anchors and on the existence of extended flexible segments. Our results demonstrate that structural and dynamical NMR parameters (cumulative Δ*δ*, SSP, temperature coefficients, relaxation time, hetNOE) combined with MD simulations can be used to build a structural model even if, due to high flexibility, the classical solution structure calculation is not possible.

## Graphical abstract

**Fuzzy in focus**: The presented hybrid NMR/MD approach indicates the disorder‐to‐order transition of p53TAD^1–60^ upon interaction with S100A4. p53TAD^1–60^ remains highly flexible, forms a clamp‐like “fuzzy” complex with three helical regions, and residues L26, M40, W53 are anchored to S100A4.

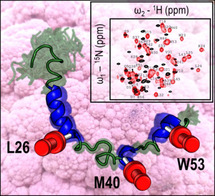

Characterizing the structural and dynamical details of highly flexible proteins/protein complexes is one of the major challenges for structural biologists. In order to characterize and understand this conformational flexibility, promising results seem to be obtained by strategies based on hybrid approaches. In our study we intend to apply such a combined NMR/MD method by investigating the challenging system formed by two biologically relevant proteins: the intrinsically disordered p53TAD and S100A4.

p53 is a key transcriptional factor that regulates several cellular processes and it is at the same time a potential therapeutic target.[^1^, ^2^] p53 plays a central role in regulating the cell cycle, triggering cell death in the nucleus and even in the cytosol.^[3]^ In its functional state the 393‐residue protein is a homotetramer with several functional domains. We focus on the N‐terminal transactivation domain (TAD; 1–60) containing subdomains TAD1 (1–41) and TAD2 (42–60).[^4^, ^5^] It is well known that many p53 interactions that are involved in the basal transcription machinery and transcriptional regulation are mediated via one or both TAD regions.[^6^, ^7^, ^8^, ^9^] Regarding structure – based on NMR, SAXS and fluorescence correlation spectroscopy methods – the TAD in apo form is intrinsically disordered and contains two regions with nascent helicity (19–25 and 47–53).[^10^, ^11^, ^12^] The class of intrinsically disordered proteins (IDPs) is characterized by highly flexible molecules with no stable secondary structure. As a consequence, these proteins/protein regions will present many conformers in solution under physiological conditions, some of them containing transient secondary structural elements, called pre‐structured motifs (PreSMo). The PreSMos can play important roles upon binding by frequently exhibiting a disorder‐to‐order transition.^[13]^ A large number of structural studies were conducted with the full length TAD or selected TAD regions in complex with various proteins[^14^, ^15^, ^16^, ^17^, ^18^, ^19^, ^20^, ^21^, ^22^, ^23^, ^24^, ^25^, ^26^, ^27^, ^28^, ^29^] (collected in Table 1 with the corresponding structures deposited in the Protein Data Bank with PDB ID). In all enumerated cases the disorder‐to‐order transition of TAD1 and/or TAD2 was detected, where the implicated subdomains formed stable helical structures upon binding to target proteins. At the same time in the bound form the regions that remained disordered could exhibit fast exchange among multiple conformations, forming so called “fuzzy” complexes.[^30^, ^31^] Due to these characteristics structure determination is challenging and the classical crystallographic methods will fail in most cases. As an alternative, NMR spectroscopy can be used to assess the solution structure with the assumption that enough structural constraints can be collected. In the case of sparse number of distance constraints, several other NMR parameters (SSP values, temperature coefficients, relaxation time data, heteronuclear NOEs) can be helpful; moreover in combination with proper MD simulations it is possible to build a solution structure.

**Table 1:** p53TAD complexes available in the literature with their PDB IDs, the partner proteins, the TAD region lengths and investigation method.

PDB ID	Partner	p53TAD region	Method
1YCQ	Mdm2	17–27	X‐ray diffraction^[14]^
2Z5S	Mdm4	17–27	X‐ray diffraction^[15]^
2Z5T	Mdm4	17–27	X‐ray diffraction^[15]^
3DAB	Mdm4	17–27	X‐ray diffraction^[16]^
4HFZ	E3 ubiquitin‐protein ligase Mdm2	17–27	X‐ray diffraction^[17]^
3DAC	Mdm4	17–28	X‐ray diffraction^[16]^
1YCR	Mdm2	17–29	X‐ray diffraction^[14]^
2MWY	Mdm4	15–29	NMR^[18]^
2L14	CBP	13–61	NMR^[19]^
5HP0	CBP	29–60	NMR^[20]^
2B3G	hRPA70	33–56	X‐ray diffraction^[21]^
2MZD	histone acetyltransferase	35–49	NMR^[22]^
2RUK	TFIIH	41–62	NMR^[23]^
2GS0	TFIIH	20–73	NMR^[7]^
2K8F	p300	1–39	NMR^[24]^
5HOU	fusion protein	1–61	NMR^[20]^
2LY4	HMGB1	1–93	NMR^[25]^
5HPD	fusion protein	2–62	NMR^[20]^
6T58	S100A4Δ8	17–56	X‐ray diffraction^[26]^

A physical and functional interaction between the metastasis related S100A4 protein and p53 was suggested for the first time by Grigorian et al.^[32]^ and was further investigated in vitro.^[33]^ S100A4 is a member of the vertebrate specific small, (10–20 kDa) EF‐hand containing Ca^2+^ binding, mostly homodimer protein family^[34]^ that plays pathological roles in tumor metastases and in inflammatory diseases.^[35]^ The Ca^2+^‐bound active form contains a hydrophobic binding pocket capable of binding to target proteins. Regarding p53 as the interaction partner, a few clinical studies have been performed where the expression level of p53 and S100A4 is determined in the same environment. A strong inverse correlation between S100A4 and p53 has been shown by immunohistochemistry in lung adenocarcinoma, suggesting that the level of S100A4 is higher in wild‐type p53 tumors.^[36]^ A trend toward inverse correlation between S100A4 and p53 was also shown in a breast cancer cohort, where a higher level of S100A4 was found to be a negative prognostic factor.^[37]^ Thus, the importance of this complex lies in its potential role in the survival of cancer cells.[^32^, ^38^, ^39^] Despite all these, only limited structural information is available. The latest effort by X‐ray crystallography was successful only if a shorter p53TAD^17–56^ segment was covalently bound to the N terminus of a C‐terminally truncated S100A4Δ8 in the presence of a crystallization chaperon protein also covalently linked to the complex.^[26]^ In the present work we modeled the solution structure of this complex using full length p53TAD^1–60^ and wild‐type S100A4.

We present how a combined NMR/MD approach can highlight the atomic level solution structure and structural dynamics information of a difficult to crystallize “fuzzy” complex using non‐tagged proteins. The precise molecular details of the S100A4‐p53 interaction aim to clarify the function and the effects of this association.

## Results and Discussion

### Structural NMR parameters: Free p53TAD^1–60^ shows a disorder‐to‐order transition upon binding to S100A4

**Figure 1:**
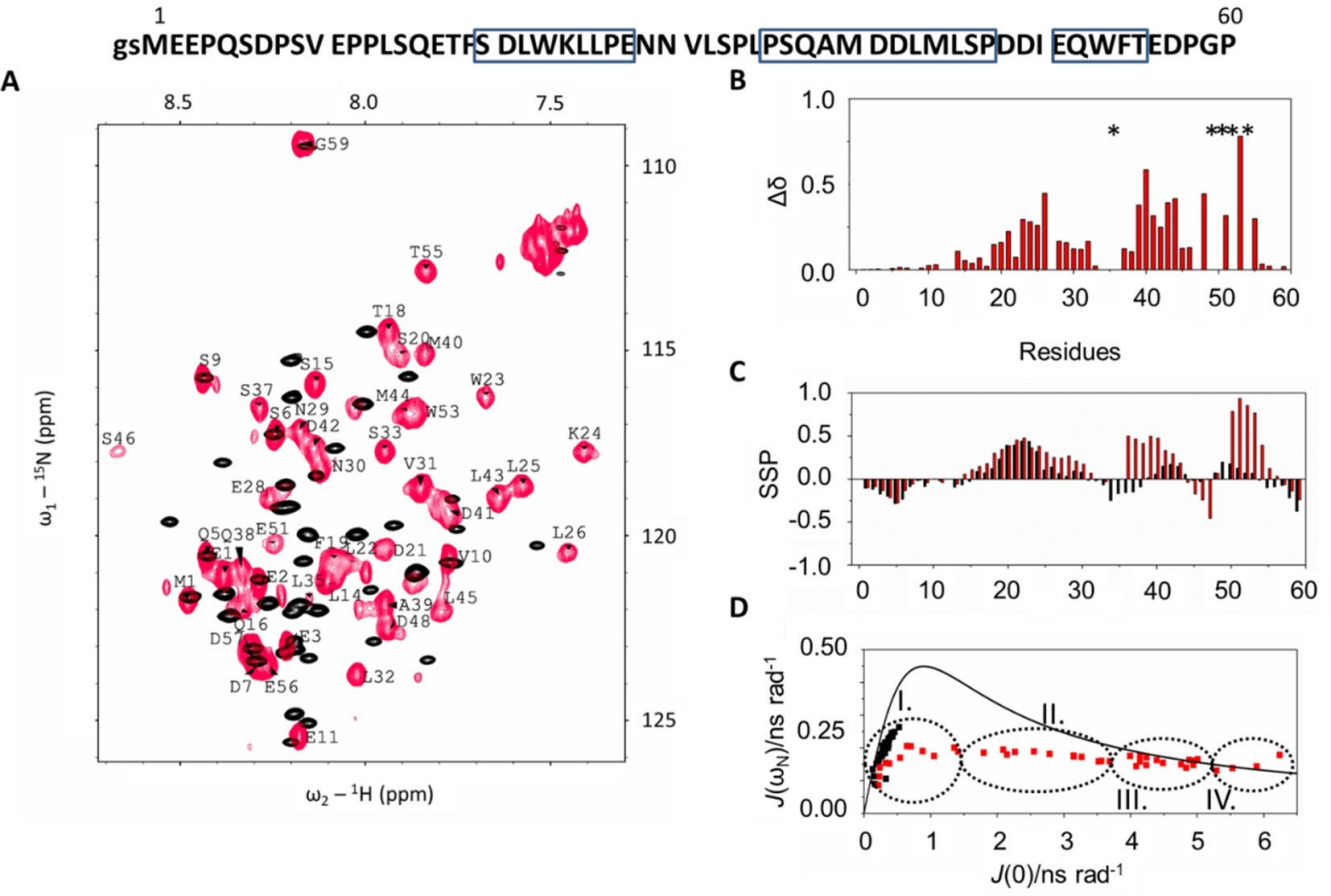
Top: amino acid sequence of p53TAD^1–60^, boxes represent the regions undergoing structural changes. A) ^1^H,^15^N HSQC spectra at 313 K and 700 MHz of ^15^N‐labeled p53TAD^1–60^ (black) and p53TAD^1–60^ in complex with unlabeled, Ca^2+^‐loaded S100A4 (red, with assignment). B) Binding information: cumulative Δ*δ* chemical shift changes of p53TAD^1–60^ resonances upon S100A4 binding. Residues broadened below the detection limit are represented by an asterisk. C) Structural information: secondary structure propensities (SSP) for free (black) and complexed (red) p53TAD^1–60^. D) Backbone dynamics results: reduced spectral density mapping *J*(ω_N_) vs *J*(0) for free (black) and complexed p53TAD^1–60^ (red).

Structural information is obtained from chemical shift values, thus resonance assignment based on triple resonance measurements, for both free and bound p53TAD^1–60^ (deposited as BMRB ID: 27597) was performed. In the case of the apo form, the results can be compared to available literature data of the p53TAD^1–93^ segment, investigated earlier under slightly different experimental conditions (BMRB ID: 17760),^[40]^ and no significant differences are detected. The complex formation between ^15^N‐labeled p53TAD^1–60^ and unlabeled Ca^2+^‐S100A4 is monitored by chemical‐shift mapping from ^1^H,^15^N HSQC spectra. The spectra for both free and bound p53TAD^1–60^ exhibit the characteristic feature of IDPs (Figure 1A), namely, a reduced chemical‐shift dispersion particularly pronounced in the ^1^H dimension. p53TAD^1–60^ residues involved in complex formation are revealed by calculating the variation of individual chemical shifts upon complex formation represented by the cumulative Δ*δ* values.^[41]^ (Figure 1B) Considering 0.15 as an arbitrary limit for reference – as being non‐perturbed residues – the S20–N29, A39–M44, D48–T55 regions are indicated to be primarily involved in binding. Residues L26, M40, W53 show the highest values, meaning these environments are the mostly perturbed ones by the interaction. Note that, for several residues (especially in the D48–T55 region) values are missing, as these resonances are broadened below detection limit. The line width analysis in the ^1^H dimension reveals that while for the free protein values hardly deviate from the average, in the complex significant variations are detected in the 40–200 Hz range (Figure S1 in the Supporting Information).The interaction causes a molecular size increase from 7 to 31 kDa (S100A4 dimer: ∼24 kDa) slowing down the tumbling rate, thus the peaks involved in binding experience an increased line broadening, whereas both N and C termini remain unperturbed indicating that these segments are not directly bound to S100A4.

SSP scores, determined from the H^α^, C^α^, C^β^ chemical shift values, are used to obtain structural information (Figure 1C).^[42]^ The free p53TAD^1–60^ is characterized by transient helical propensities in the F19–K24 and to a minor extent in the D41–L43, D49–E51 regions. Due to complex formation, the helical tendency is expanded in the T18–N29 part. The high SSP values in the P36–D42 and I50–F54 regions indicate the presence of newly formed helices. All these data show a folding upon binding transition for the disordered TAD segment. To support the notion of helix formation in the three regions indicated by SSP values, we carried out detailed analysis to determine new hydrogen bond networks, and specific structural proximities accompanying the complex formation.

Temperature coefficients calculated from the chemical shift variations of the ^1^H,^15^N HSQC resonances (Figure S2) are indicators of hydrogen bonds and fast exchange processes. In aqueous solution amide environments involved in intramolecular hydrogen bonding show values above −4.5 ppb/K, whereas values below −9 ppb/K imply residues involved in fast exchange.^[43]^ For free p53TAD^1–60^ values around −9 ppb/K are found for the Q5–D7, E11–L14, L32–S33 segments and at the E28, D57 positions – all belonging to the disordered regions, thus engaged in fast exchange processes. A slight tendency towards hydrogen bonding is observed in the K24–L25 region indicating transient helicity. In the complexed form the fast exchange is maintained for the already enumerated environments marking the unstructured regions. Hydrogen bonding is observed in the first helical part besides K24–L25 also for S20–D21, but it is more pronounced for most residues in the A39–L45 range and for T55, thus supporting the complexation induced helix formation.

Distance constraints that can generally be used for structure calculation are the NOE crosspeaks between proton environments in spatial proximity of maximum 5 Å. The structure‐specific intraresidual crosspeaks were intended to be obtained from 3D ^1^H,^15^N HSQC‐NOESY spectra. While peak intensities of the disordered regions are satisfactory, due to the previously discussed signal broadening affecting the binding segments – mostly the third helical region – only a very limited number of characteristic helical (*i*; *i*+2) and (*i*; *i*+3) NOE data can be extracted. This makes structure calculation impossible, however, the collected ^1^H−^1^H distances supported by strong NOE crosspeaks – concentrated mostly in the second helical segment (Table S1) – are sufficient to support building the starting models of MD simulations (which are then run constraint‐free) and can be used to verify the results.

### Backbone dynamics: Differences between the free and bound p53TAD^1–60^ behavior

Backbone dynamics is typically revealed from *R_1_*, *R_2_* ^15^N relaxation rates and ^1^H,^15^N heteronuclear steady‐state NOE data (Figure S3). Comparing the free and complex states of p53TAD^1–60^, *R*_1_ longitudinal relaxation rates follow a relatively uniform distribution in both cases. The unstructured N terminus shows almost identical values but in the bound form slightly decreased values are observed. On the other hand, the *R*_2_ transverse relaxation rates increase notably upon complex formation indicating the disorder‐to‐order transition. In the free form a uniform distribution around 2 s^−1^ is observed while in the complex these values vary largely in the 1.4–25 s^−1^ range, with particular increase in the E17–S33, S37–T55 helical propensity segments, presenting the highest values for the W23–L25, D42–L45 and E51 environments. The steady‐state ^1^H,^15^N heteronuclear NOE data also indicate that the molecule is more structured in the complexed form. Again the highest numbers belong to segments W23–L26, D41–S46, and environments E51, T55 (several residues are undetected in this domain due to previously mentioned line broadening). An in‐depth analysis of these relaxation data in the case of IDPs can be carried out by the reduced spectral density mapping analysis,^[44]^ calculating the spectral density functions *J*(0), *J*(ω_N_) and *J*(ω_h_). *J*(0) refers to the probability of slow motions in the rotational correlation function (mainly in rigid domains), *J*(ω_h_) to the fast motions (flexible regions) and *J*(ω_N_) to the intermediate regime. The mapping is represented by the *J*(0)−*J*(ω_N_) graph (Figure 1D). In our case a clear distinction is observed: for free p53TAD^1–60^ all residues fall in the small *J*(0) range close to the single motion curve in accordance with high mobility and disordered structure. In the complex a wide spread along *J*(0) can be seen allowing the clustering of residues into four groups. Group I includes the highly mobile residues of the termini: M1–E11, D57–G59, and residue L35. Group II contains mobile residues L14–T18, N29–S33, D48, E56, whereas Group III consists of residues belonging to helices according to the SSP values discussed previously: S20–E28, S37–S46, T55. The most rigid residues are in Group IV: W23, K24, L32, E51 and these environments might be involved in conformational exchange processes as well.

Taken together, all these NMR parameters describe the structure of p53TAD^1–60^ in the complex as having highly mobile long N (M1–Q16) and short C termini (E56–P60) regions that are not involved in the interaction as they maintain the behavior detected also in their free state. Three detached helices are formed, one in TAD1 (T18–N29), the second spans through TAD1 and TAD2 (P36–P47), while the third is located in TAD2 (I50–T55). The helices are connected by relatively dynamic loops (N30–L35 and D48–D49). Possible conformational exchange is shown for residues belonging to the first helix (W23, K24) and for L32 situated in the flexible loop.

### p53TAD^1–60^ causes an asymmetry in the Ca^2+^ loaded S100A4 dimer

To be able to build a realistic starting model for MD calculations we need to define the binding groove of the Ca^2+^‐loaded S100A4. In the available structures of S100A4 complexes, an asymmetric, so called “hugging interaction“ has been observed: the protein chain of the interacting partner winds around the S100A4 dimer, and depending on how tight the interaction is, the equivalence of the two monomer chains will be diminished.^[45]^ The resulting complexes exhibit an asymmetric structure, as the two canonical binding grooves – formed by the L2 loop and helices H3, H4 – become different upon interaction with the partner.[^46^, ^47^] In order to test whether this behavior is valid/maintained also in the case of interaction with p53TAD^1–60^ we performed the chemical shift mapping (Figure S4) with ^15^N‐labeled S100A4 and unlabeled p53TAD^1–60^. Due to the fuzzy nature of considerable segments in p53TAD^1–60^, it is not surprising that the non‐equivalence of the two S100A4 monomer chains is only visible in a few locations, that is, the number of double peaks for a given residue (belonging to chain I and II, respectively) is restricted (see Figure S4 for explanation). Even though the cumulative chemical shifts are small, it is clearly observable that L2 loop and helices H3, H4 suffer the most pronounced perturbation, as expected. Moreover, broadened resonances are detected mainly in the binding region of S100A4, indicative of an intermediate exchange between the free and bound S100A4 states. The relatively high number of peaks showing intermediate exchange also suggests the flexibility of the complex. Taken altogether, p53TAD^1–60^ binding represents another example of an asymmetric S100A4 complex formation and this NMR information helps in building the initial structure, and the contact surfaces in MD simulations.

### Molecular dynamics simulations

**Figure 2:**
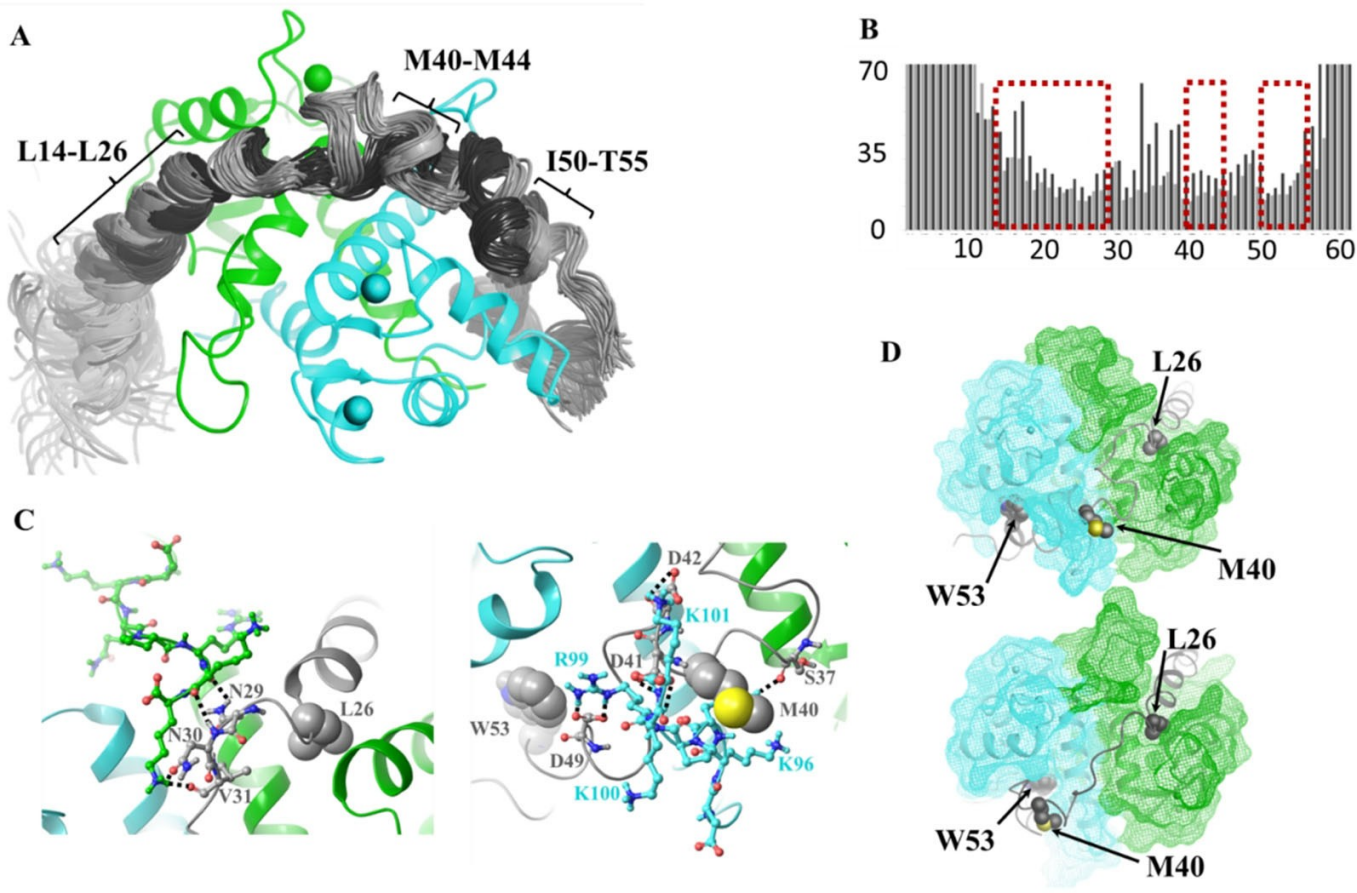
A) Structure of the MD‐derived complexes. Cluster mid‐structures are shown for both Model A (dark gray) and Model B (light gray) accounting for 90 % of all snapshots of the last 300 ns of the simulations. For clarity, the S100A4 dimer is only shown in a single copy in green (monomer I) and cyan (monomer II). B) Calculated *B*‐factors from the MD models. The boxes indicate the position of the main helices. C) Examples of the H‐bonds formed during the MD simulations between the C‐terminal 95–101 segments of S100A4 (chains A (green) and B (cyan) are shown explicitly) and p53TAD^1–60^. Interacting and anchoring residues of p53TAD^1–60^ are also highlighted, D) Anchoring positions. The position of the three residues that undergo the largest chemical‐shift change on complexation: L26, M40 and W53 are labeled.

All the above presented NMR parameters were used in setting up four different starting models for the MD simulations. Following the incorporation of the detected transient NOE distance constraints in the model building step, the calculations were carried out constraint‐free. The resulting models are expected to fulfill as many NOE constraints as possible while at the same time maintaining the structural characteristics of the complex. Two characteristically different arrangements (Model A and Model B) are selected to represent the scope of variations (Figure 2A). Both models fulfill the experimentally determined NOE constraints (Table S1), and contain two stable helices: L14–L26 and I50–T55 in full accordance with the NMR experiments. In some structures of the equilibrium trajectories the D7–P13 region is also helical, but it is situated in the solvent and wiggles considerably. This is reflected in the obtained very high *B*‐factors that correlate well with the high mobility of this region detected by the NMR backbone dynamics (Figure 2B).

In Model B, the C terminus of p53TAD^1–60^ is shifted toward the solvent and the C‐terminal tail of chain II from S100A4 is flipped to an open conformation causing a coupled restructuring and an increase of helicity in the middle part of p53TAD^1–60^. In this model – in addition to the N‐ and C‐terminal helices – the M40–M44 segment is also helical (Figure 2A).

The unstructured C‐terminal loops of both S100A4 monomers – though remaining highly solvent exposed – participate in complex formation: in both models the R99–K101 segment in chain I forms H‐bond contacts with D21, E28, N30, V31 of the p53TAD^1–60^ partner; while the C‐terminal Q97, K100, K101 part of chain II interacts with M40, D41, D42 from the p53TAD^1–60^ side in Model A, and in Model B it is immersed in the solvent. (Figure 2C).

Residues of p53TAD^1–60^ showing the largest chemical shift changes upon complex formation in the NMR experiments – L26, M40 and W53 – proved to be the major anchors of the MD‐derived ensembles (Figure 2D), demonstrating characteristically low *B*‐factors (Figure 2B). L26 and W53 are immersed into the same hydrophobic pocket of the monomeric units of the S100A4 dimer (formed by residues L42, F45, L58, L62, V77, F78, C81 and I82) while M40 is situated on the ridge connecting the two monomers. It is also notable that the S100A4 regions creating the binding pocket for the anchoring p53TAD^1–60^ residues (helices H3 : 50–63 and H4 : 72–87) were also recognized by NMR chemical shift mapping showing higher Δ*δ* values in the T50–Q56 and E74–A83 regions (Figure S4B).

### Comparison of the available p53TAD structures and dynamics

**Figure 3:**
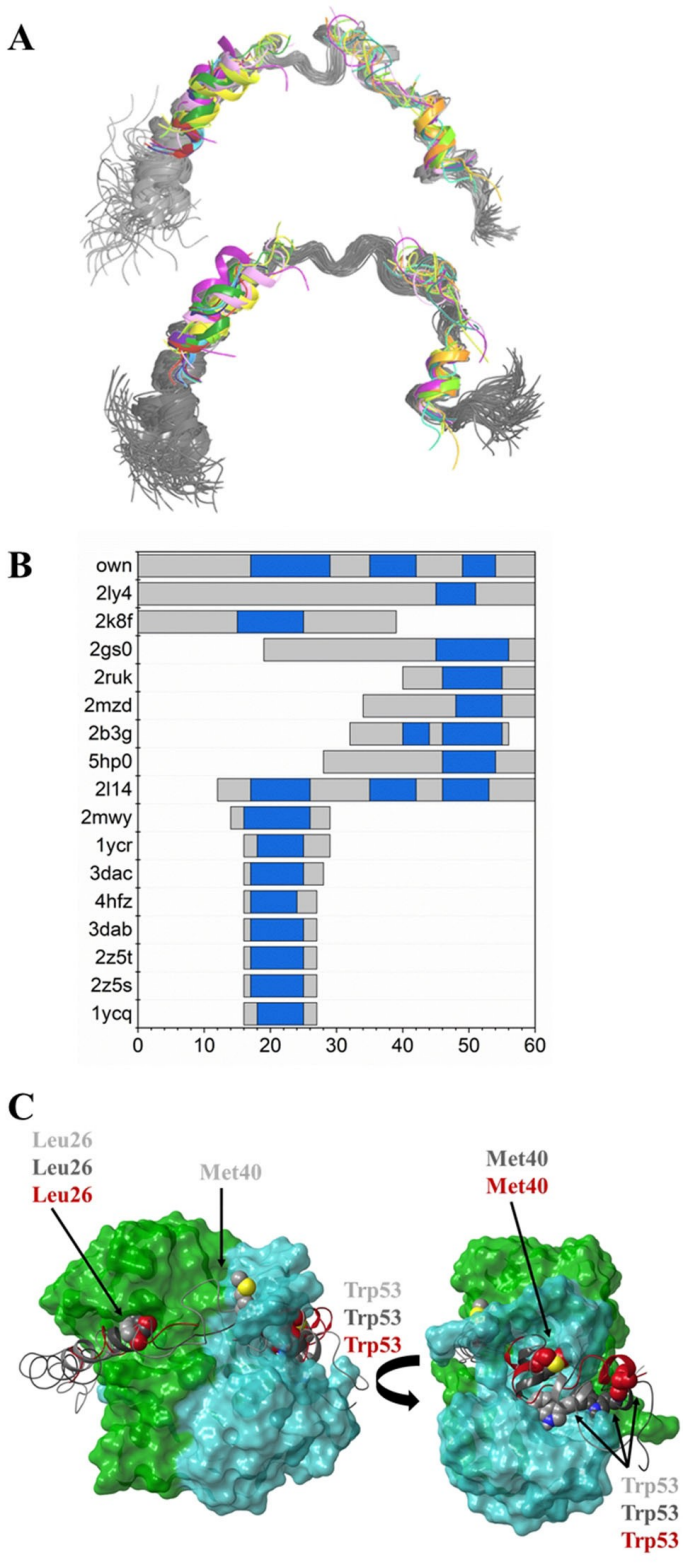
A) p53TAD structures (from Table 1, in color) in various complexes overlaid independently on the MD‐derived conformational ensembles of the present work (Model A in light gray, Model B in dark). B) Secondary structures of p53TAD in the above complexes. The length of the investigated fragment is shown in light gray, blue parts denote the helical regions. C) Overlaid structures of p53TAD^1–60^ from the of the NMR/MD‐derived p53TAD^1–60^–S100A4 complex (Model A in light gray, Model B in dark) and the crystallographically determined p53TAD^17–56^ segment of the p53TAD^17–56^–S100A4Δ8 complex in red, showing similar anchor points on the surface of the S100A4 dimer (shown in cyan/green in a single copy and based on the MD simulation).

p53TAD segments of various lengths including either one or both TAD regions were subject to interaction studies with various partners (Table 1). These structures – determined either by X‐ray crystallography or NMR spectroscopy – are shown superimposed in Figure 3A; secondary structural elements are highlighted in Figure 3B. As observed, in most examples the behavior of TAD1 is investigated, and for all cases helix formation encompassing the (T18–L25) region is detected. The TAD1 region is the primary binding site for Mdm2 and Mdm4 and folds into an amphipathic helix (T18–L25) upon complex formation.[^14^, ^15^, ^16^, ^17^, ^22^] This helical TAD1 structure is also detected in complexes formed with p300^[25]^ and CBP.^[18]^ Regarding the behavior upon complex formation of the TAD2 region a short helix at the C terminus (P47–T55) is detected in several cases.[^7^, ^16^, ^18^, ^22^, ^23^, ^25^] Residues P47–T55 fold into a helical structure when TAD2 binds to TFIIH.[^7^, ^23^] Binding to CBP is also dominated by interactions with TAD2. Binding to hRPA70 causes TAD2 to fold into two short helices, located between D41–M44 and P47–T55.^[21]^ Few examples exist that encompass both TAD1 and TAD2 regions. Upon interaction of p53TAD^13–61^ with CBP three helices are formed in segments T18–L26, P36–D42 and P47–W53.^[19]^ A short helical region was observed between residues S46 and E51 in the p53TAD^1–93^–HMGB1 complex with a disordered region showing helical tendency in the F19–L26 region.^[25]^ The present study incorporating both TAD1 and TAD2 parts detects the formation of a third, short helical motif.

Although structural insights are relatively abundant, less information is available about the motional description of bound p53TAD segments. In this respect Vise et al.^[29]^ investigated the dynamics of the p53TAD^1–73^–hRPA70^1–168^ complex by relaxation measurements and for data analysis the reduced spectral density mapping method was used as well. Comparing to our present results – even though those measurements were performed at 298 K – a similar variation and change in the *R*_2_ rates upon complex formation was detected, however, only in the (D41–D57) region of p53TAD^1–73^. In contrast, we detected the increase of transverse relaxation rates also in the N‐terminal part starting from residue L14. In their case only the D42–D57 region showed elevated values of *J*(0) with respect to the free form, while we detected an increase in *J*(0) for almost the entire protein (L14–G59 region). It is worth noting that due to the line‐broadening effect, a fourfold excess of p53TAD^1–73^ was used, thus meaning it could not be entirely saturated with the partner causing a partial change both in structure and dynamics. Our results refer to a 1 : 1 ratio of the interaction partners and we noticed the disordered‐to‐ordered transition in nearly the entire protein excluding the N‐terminal M1–P13 region. Steady‐state heteronuclear NOE measurements were performed for p53TAD in complex with TAZ1 and TAZ2 domains of CBP. (*R*_1_ and *R*_2_ rates were not determined).^[20]^ The change in hetNOE values were similar to our results: TAD1 (Q16–L25) becomes helical in complex with both TAZ1 and TAZ2 subdomains, while TAD2 is only partially helical (P47–W53 region in complex with TAZ1 and P47–T55 in complex with TAZ2).

In conclusion the slight differences in the position and length of the detected/formed helical regions can be the reason of the binding specificity of p53, determined also by the interaction partner.

### The p53TAD‐S100A4 structures

Finally, we compare the recently solved crystal structure of the p53TAD^17–56^–S100A4Δ8 complex with our solution structure of p53TAD^1–60^–full‐length S100A4 complex (Figure 3C). In order to be able to crystallize the “fuzzy” system, several modifications were carried out: a shortened p53TAD^17–56^ was covalently liked to a truncated S100A4Δ8 construct also fused to a non‐EF hand Ca^2+^ binding annexin A2 which was utilized as a crystallization chaperon.^[26]^ In the crystal structure three short segments showed helical conformation: T18–L25, S37–D42 and P47–W53, whereas the V31–L35 segment could not be resolved in the electron density, thus indicating high conformational variability in this region. Residues belonging to L22–P27 of the first helix as well as S46 and I50 have lower *B*‐factors than their surroundings, thus they seem to be the primary interaction sites. Important hydrophobic contacts are formed by residues F19, L22, W23, L25, L43, L45, I50, and W53. In our current combined NMR/MD based model p53TAD^1–60^ shows mobile N‐terminal (M1–P13) and C‐terminal (E56–P60) regions that are not involved in complex formation. Three helices are displayed in regions: T18–N29, P36–P47 and I50–T55 connected by relatively dynamic segments–forming loops. These structural features are supported by chemical shift values, temperature coefficients, and backbone dynamics analysis. The first and the third helical regions mostly overlap in the structures determined by the two different methods. Importantly, these helices are also present in other TAD complexes.[^14^, ^15^, ^16^, ^17^, ^18^, ^19^, ^20^, ^21^, ^22^, ^23^, ^24^, ^25^] Interestingly, the second helix (P36–D42) is visible only in the NMR/MD model and not in the crystal structure, however it is also observed in the complex of p53TAD with CBP. The appearance of this element can be a consequence of the interaction of TAD with the C terminus of the S100A4. This is a likely important difference between the solution and the crystal structure of the chimeric construct. MD simulation snapshots on Figure 2C indicate that H‐bonds are formed in both models between the segments of both S100A4 chains and p53TAD^1–60^, highlighting the importance of the C‐terminal S100A4 tail in the interaction. Moreover, NMR/MD investigations prove that S100A4 exhibits an asymmetric binding to p53TAD^1–60^ as well.

## Conclusion

Results obtained from solution NMR structural and dynamical studies combined with MD simulations indicate that both TAD1 and TAD2 subdomains of p53TAD^1–60^ undergo disorder‐to‐order transition upon complex formation and mediate the binding to S100A4. Complex formation of the p53TAD^1–60^ comprising both TAD1 and TAD2 domains might follow the “two point interaction” model described for cases where two distinct parts from p53 contact distinct areas of the partner (in the present case S100A4)^[21]^ and a “fuzzy” complex is formed. The involvement of hydrophobic interaction in stabilizing the complex via a Ø−X−X−Ø−Ø segment with Ø representing the hydrophobic residue is fulfilled for both F^19^SDLW^23^ and I^50^EQWF^54^ helices.^[10]^ Residues W53 and F54 are important for binding, moreover L26 and M40 contribute as key anchoring positions. The more pronounced helix at the C terminus might play a primary role in complex formation. These results suggest that formation of a conserved local structure is a feature of p53 recognition. The helical segments formed in TAD1 and TAD2 regions function as clamps, and are connected by a disordered flexible linker. Such clamp models have been observed for several other complexes with an IDP as one interaction partner (Table S2) providing flexibility and adaptability in molecular interactions. For our system the obtained MD models shed light on the fuzziness of this complex, especially in the E28–Q38 segment and the terminal regions of p53TAD^1–60^. The C terminus contains a short helix running from residues I50–E56, but the helix and the following unstructured loop is shifted about in the broad binding channel formed by the S100A4 dimer. The C‐terminal tail of S100A4 is shown to be important for stabilizing the complex.

The p53TAD‐CBP complex was also described as a “fuzzy” complex (ID: FC0084 in the FuzDB Fuzzy Complexes Database)^[48]^ – enabling simultaneous binding of two interaction motifs to the target protein. The intrinsic disorder of the TAD region provides the flexibility to form ternary complexes with MDM2 or CBP/p300 domains and makes it possible to bind with enhanced affinity through such clamp‐like interactions. A similar conformational heterogeneity has been described for the intrinsically disordered myelin basic protein (MBP^145–165^) in interaction with calmodulin,^[49]^ another Ca^2+^‐binding protein (FuzDB ID: FC0082). This fuzziness provides the plasticity needed for interactions with numerous different targets and enables the relevant recognition processes to take place.

In conclusion, our results represent an example of how to combine NMR and MD in order to obtain realistic information about complex systems that represent challenges for crystallization; and also when even in solution studies obtaining crucial information is not too straightforward. The atomic level structural characterization of the p53TAD–S100A4 complex now by two distinct approaches can contribute to the identification of p53 inhibitor molecules and to the development of biosensors based on S100A4 binding. Our findings emphasize that, despite the difficulties, structural studies of fuzzy complexes by such hybrid methods can be crucial to understanding the complex functions of hub proteins similar to p53. Disorder information for such protein complexes can be employed in new strategies of drug discovery, for determination of protein‐protein interaction inhibitors as well.

## Experimental Section

**Protein expression**: Human p53TAD^1–60^ (UniProt code: P04637) was cloned into a modified pGEX vector (pETARA) containing an N‐terminal TEV‐cleavable glutathione S‐transferase (GST) tag. The wild‐type human S100A4 (UniProt code: P26447) was cloned as previously described.^[45]^

p53TAD^1–60^ and S100A4 constructs were expressed in *Escherichia coli* BL21(DE) cells. Transformed cultures were grown in lysogeny broth (LB) supplemented with 100 μg/mL ampicillin at 37 °C until the optical density at 600 nm reached 0.8. Expression was induced with 0.5 mM isopropyl β‐d‐1‐thiogalactopyranoside (IPTG) at 28 °C for 4 h in the case of GST‐fusion p53 peptides and at 37 °C for 3 h in the case of S100A4. Cells expressing the ^15^N‐ and ^13^C,^15^N‐labeled p53TAD^1–60^ peptides were transferred into a minimal broth containing 50 mM Na_2_HPO_4_, 20 mM KH_2_PO_4_, 8.5 mM NaCl, 1 mM CaCl_2_, 2 mM MgSO_4_, 18.7 mM NH_4_Cl and 22.2 mM glucose used in ^15^N/^13^C‐labeled form, respectively, in accordance with necessity before inducing them with IPTG. Pelleted cells were disintegrated by ultrasonication in a buffer containing 20 mM Tris, pH 8, 300 mM NaCl, 0.1 mM tris(2‐carboxyethyl)phosphine (TCEP), and 1 mM phenylmethanesulfonyl fluoride (PMSF). Cell lysates were clarified by centrifugation at 48 000 *g*. Supernatant of S100A4 expressing cells was applied to Ni^2+^ affinity chromatography column using Profinity IMAC resin (Bio‐Rad) with 20 mM Tris, pH 8, 300 mM NaCl, 0.1 mM TCEP as wash buffer and were eluted with the wash buffer complemented with 250 mM imidazole. Supernatant of p53TAD^1–60^ peptides expressing cells were loaded onto Protino Glutathione Agarose 4B resin (Macherey‐Nagel). After thorough wash with the lysis buffer the GST‐fusions were eluted using the washing buffer complemented with 10 mM reduced glutathione. GST and His_6_ tag were eliminated using TEV protease at room temperature overnight. After complete cleavage, GST was removed from solution by heat denaturation followed by centrifugation. p53TAD^1–60^ fragments were further purified by reversed‐phase HPLC on a Jupiter 300 C5 column (Phenomenex). The p53TAD^1–60^ containing fractions were lyophilized and stored at −20 °C. S100A4 were applied to phenyl‐Sepharose 6 resin column (GE Healthcare), the storage buffer was complemented with 1 mM CaCl_2_ before chromatography, washed with 20 mM HEPES, pH 7, 50 mM NaCl, 0.5 mM CaCl_2_, 0.1 mM TCEP to change the buffer of the sample, and eluted with the wash buffer supplemented with 5 mM EGTA. S100A4 was concentrated using Amicon Ultra‐15 Centrifugal Filter Units and stored at −80 °C.

**NMR measurements**: All samples were prepared to have ca. 1 mM (^15^N‐ or ^13^C,^15^N‐labeled) protein concentration, containing as well 20 mM MES, 20 mM NaCl, 10 mM TCEP, 10 mM CaCl_2_, 3 mM NaN_3_, 10 % D_2_O, 1 % DSS at pH 6.0. Chemical shift mapping was done in steps titrating the labeled protein with the unlabeled partner (p53TAD^1–60^, or Ca^2+^ loaded S100A4 dimer) in steps covering the 30–115 % titration range.

NMR measurements were performed on a 16.4 T Bruker Avance III spectrometer, operating at 700.17 MHz for ^1^H, 176.05 MHz for ^13^C and 70.94 for ^15^N, equipped with a ^1^H/^13^C/^15^N 5 mm *z*‐gradient probe at 313 K. ^1^H chemical shifts were referenced to the internal 4,4‐dimethyl‐4‐silapentane‐1‐sulfonic acid standard, whereas ^15^N and ^13^C chemical shifts were referenced indirectly via the gyromagnetic ratios.

Resonance assignment and sequential connectivities were determined for p53TAD^1–60^ from the analysis of 3D BEST type HNCA, HN(CO)CA, HNCACB, HN(CO)CACB, HN(CA)CO, and HNCO, as well as CCCONH, ^1^H,^15^N HSQC, ^1^H,^13^C HSQC measurements. ^1^H,^1^H distance constraints from transient NOE crosspeaks were assigned based on 3D HSQC‐NOESY (mixing time: 150 ms), 3D HSQC‐TOCSY (mixing time: 70 ms) spectra. Determined chemical shifts were deposited into BMRB data base (ID number: 27597). Chemical shift assignment of S100A4 was done based on 3D HSQC‐NOESY (mixing time: 150 ms), 3D HSQC‐TOCSY (mixing time: 70 ms) spectra and our previous results^[41]^ were used as well.

For backbone dynamics studies of p53TAD^1–60^ *t*_1_ relaxation time measurements were done in 10 steps with a variable delay between 0.01–2 s, while *t*_2_ relaxation time measurements were measured in 11 steps using delays between 0.03–1.36 s, in both cases one delay has been recorded twice to check data reproducibility. ^1^H,^15^N heteronuclear steady‐state NOE measurements were carried out with and without proton saturation using an 8 s relaxation delay. Relaxation times were evaluated from single exponential decays using peak intensities, and heteronuclear steady‐state NOEs were calculated from the intensity ratios of individual peaks with and without proton saturation Data were further analyzed by reduced spectral mapping method.^[44]^

Data were processed with the TopSpin program and analyzed by Sparky,^[50]^ CCP NMR^[51]^ and CARA^[52]^ softwares. Secondary chemical shift values were calculated using sequence corrected random‐coil chemical shifts.^[53]^

**Molecular dynamics simulations**: Initial models of the p53TAD^2–60^–S100A4 complex were built based on the PDB structures 3CGA and 2LNK by using the NOE constraints in energy minimization steps carried out using MacroModel of the Schrödinger Suite. MD simulations were carried out as implemented in GROMACS54, using the AMBER99SB‐ILDNP forcefield. Systems were solvated by OPC water molecules in dodecahedral boxes with 10 Å buffer. The total charge of the system was neutralized and physiological salt concentration set using Na^+^ and Cl^−^ ions. Energy minimization of starting structures (by GROMACS) was followed by sequential relaxation of constraints on protein atoms in three steps (each of 100 ps). Trajectories of 700 ns NPT simulations at 310 K and 1 bar were recorded for further analysis (collecting snapshots at every 4 ps). Clustering of conformations was carried out based on the main‐chain conformation of p53TAD^2–60^ in the last 300 ns of the simulations using a cutoff of 1.0 Å. The applied forcefield in combination with a four‐point water model (OPC waters – in this study) was shown to correctly reproduce the SAXS profiles of systems with extreme flexibility.^[54]^

## Conflict of interest

The authors declare no conflict of interest.

## Supporting information

As a service to our authors and readers, this journal provides supporting information supplied by the authors. Such materials are peer reviewed and may be re‐organized for online delivery, but are not copy‐edited or typeset. Technical support issues arising from supporting information (other than missing files) should be addressed to the authors.

Supplementary
