# Supplementary material for: Tumor‐Suppressor p53TAD1–60 Forms a Fuzzy Complex with Metastasis‐Associated S100A4: Structural Insights and Dynamics by an NMR/MD Approach
Source: Chembiochem. 2020 Jul 22;21(21):3087–95. doi: 10.1002/cbic.202000348 (PMC7689910; doi:10.1002/cbic.202000348)
Supplement: Supplementary file 1 — Supplementary [file CBIC-21-3087-s001.pdf]

ChemBioChem

Supporting Information

Tumor-Suppressor p53TAD$^{1-60}$ Forms a Fuzzy Complex with Metastasis-Associated S100A4: Structural Insights and Dynamics by an NMR/MD Approach

Erika F. Dudás, Gyula Pálfy, Dóra K. Menyhárd, Fanni Sebák, Péter Ecsédi, László Nyitray, and Andrea Bodor*

Author Contributions

E.D. Investigation:Lead; Methodology:Lead; Writing - Review & Editing:Supporting
G.P. Investigation:Supporting; Methodology:Supporting
D.M. Conceptualization:Supporting; Investigation:Equal; Methodology:Equal; Validation:Equal; Writing - Review & Editing:Supporting
F.S. Investigation:Supporting; Methodology:Supporting; Software:Supporting; Writing - Review & Editing:Supporting
P.E. Methodology:Supporting; Writing - Original Draft:Supporting
L.N. Conceptualization:Supporting; Funding acquisition:Supporting; Investigation:Supporting; Methodology:Supporting; Writing - Review & Editing:Supporting

Supporting information

Table of contents

Figure S1. ^{1}H line width values for p53TAD$^{1\text{-}60}$ (black) and p53TAD$^{1\text{-}60}$ in complex with S100A4 (red).

Figure S2. Temperature coefficient (ppb/K) values for p53TAD$^{1\text{-}60}$ (black) and p53TAD$^{1\text{-}60}$ in complex with S100A4 (red).

Figure S3. Backbone dynamics parameters (R_1, R_2, steady state HetNOE) for p53TAD$^{1\text{-}60}$ (black) and p53TAD$^{1\text{-}60}$ in complex with S100A4 (red).

Figure S4. Binding regions of S100A4 detected by chemical shift mapping. Overlay of the ^{1}H-^{15}N HSQC spectra for the free and complexed form, Δδ values and exchange regimes in the S100A4–p53TAD$^{1\text{-}60}$ complex.

Table S1. Experimentally (NMR spectroscopy) detected long range NOE constraints and MD simulations regarding fulfillment of these constraints.

Table S2. The clamp model in other protein systems with an IDP and the partner molecule.

Figure S1. ^{1}**H line width values for p53TAD$^{1\text{-}60}$ (black) and p53TAD$^{1\text{-}60}$ in complex with S100A4 (red).**

Residues broadened below detection limit are represented by an asterix.

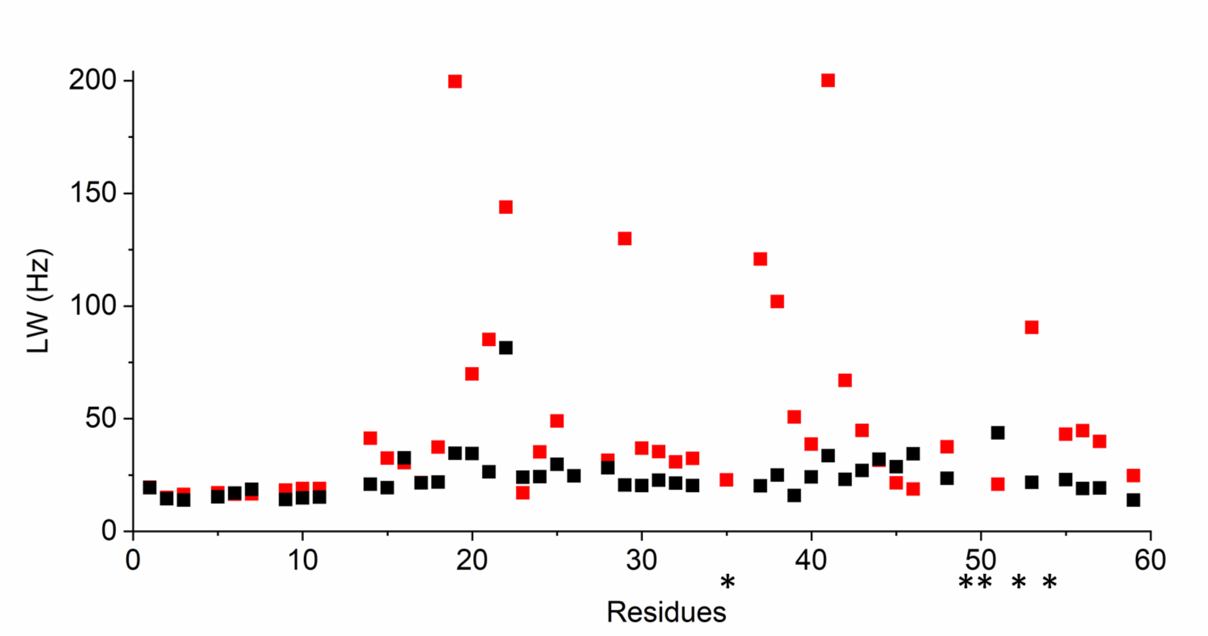

Figure S2. Temperature coefficients (ppb/K) values for free p53TAD$^{1\text{-}60}$ (black) and p53TAD$^{1\text{-}60}$ in complex with S100A4 (red).

Residues broadened below detection limit are represented by an asterix, prolines are represented by black triangles.

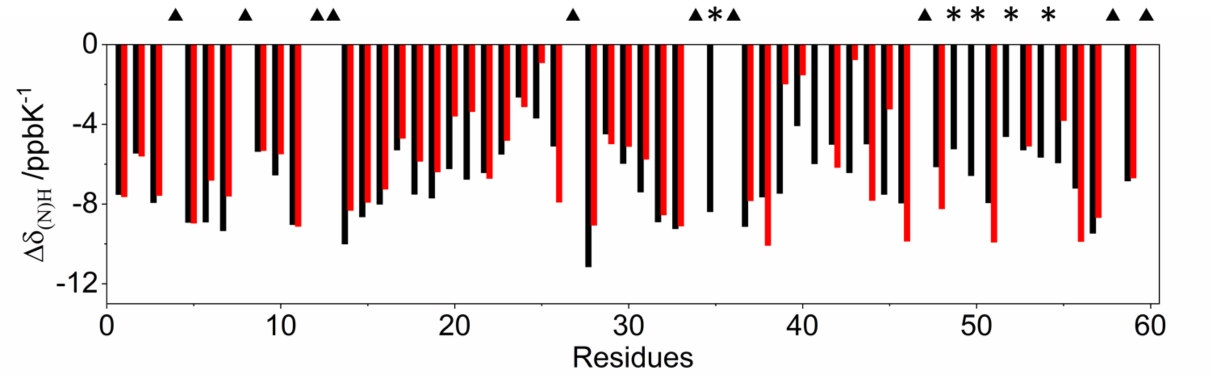

Figure S3. Backbone dynamics parameters (R_1, R_2, steady state HetNOE) for p53TAD$^{1\text{-}60}$ (black) and p53TAD$^{1\text{-}60}$ in complex with S100A4 (red).

Longitudinal (R_1), transverse (R_2) relaxation rates and steady state heteronuclear NOE values plotted against the sequence. Bars indicate the determination uncertainty, which in many cases is hardly visible. Residues broadened below detection limit are represented by an asterix.

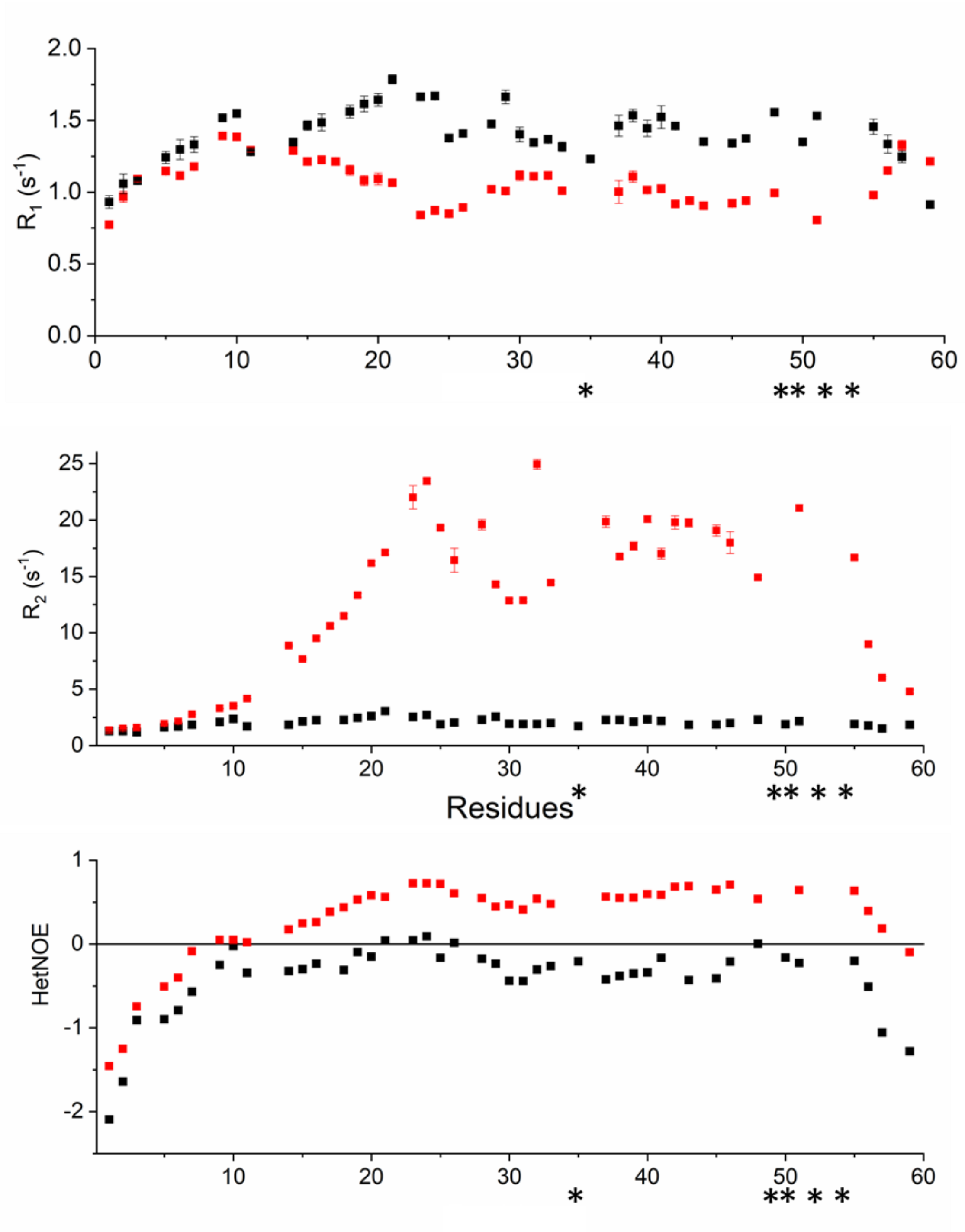

Figure S4. Binding regions of S100A4 detected by chemical shift mapping. Overlay of the ^{1}H-^{15}N HSQC spectra for the free and complexed form, Δδ values and exchange regimes in the S100A4–p53TAD$^{1-60}$ complex.

(A) ^{1}H-^{15}N HSQC spectrum of ^{15}N-labeled S100A4 in free form (red) and in complex (blue) with unlabeled p53TAD$^{1-60}$.

(B) The cumulative chemical shift changes (Δ*δ, calculated as described in the main text*) upon complex formation. In case of peak doubling – due to the non-equivalence of chain I and II of S100A4 in the complex - the resulting chemical shift changes for these environments are shown in blue and magenta. Green rectangles indicate residues broadened below detection limit upon complex formation – and no value could be calculated. Grey arrows indicate the residues that could not be detected or assigned (prolines and missing assignment due to signal overlap). The secondary structure of S100A4 is shown above the graph (helices H1-H4, loops L1-L3 and the C-terminal disordered tail).

(C) The amino acid sequence of S100A4. Colors refer to different NMR exchange regimes in the complex:

-slow exchange between the free and the bound form: the bound form gives rise to one peak, *i.e.* these environments in the two monomer chains remain equivalent (red); in the bound form the environments in the two monomer chains differ (magenta)

-intermediate exchange causes line broadening in many cases: with one peak in bound form (light green) or doubled peaks in bound form (dark green)

-fast exchange, one peak is detected, which shifts upon complex formation, or is hardly moving (black). Peaks that could not be detected or assigned are shown in grey.

We present examples for three residues belonging to different exchange regimes. Corresponding overlaid ^{1}H-^{15}N HSQC from the stepwise addition of unlabeled p53TAD$^{1-60}$ are zoomed for the chosen environments: K31, L9, G47.

K31 presents fast exchange, L9 slow exchange resulting in one peak, G47 slow exchange resulting in two peaks. The initial free (non-bound) states are red, the cca 50% addition state is purple and the final complexed state is blue.

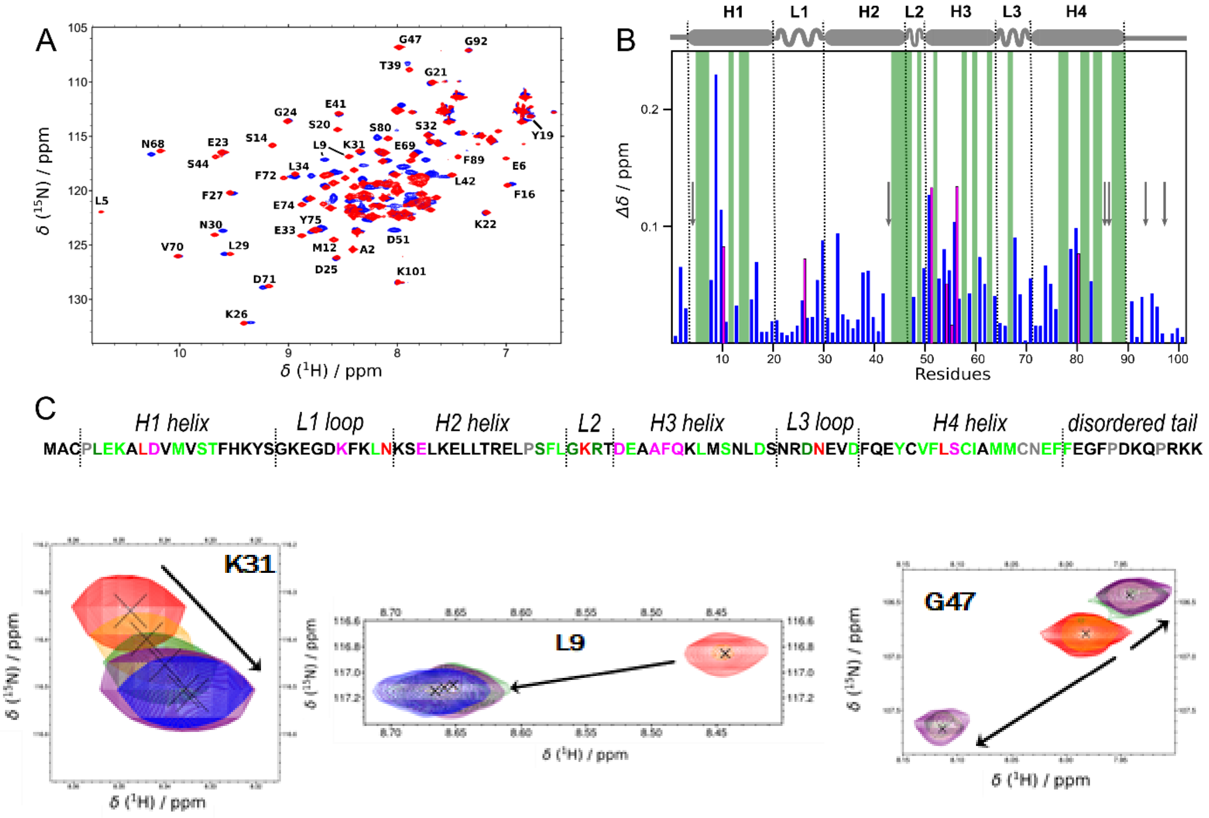

A
δ (¹H) / ppm
δ (¹⁵N) / ppm
G47
G92
T39
G21
E41
G24
S20
S80
S32
N68
E23
S14
L9
K31
E69
Y19
S44
L34
F89
E6
L5
F72
L42
F27
F16
E74
Y75
N30
E33
K22
D51
V70
L29
M12
A2
D25
K101
D71
K26
B
H1
L1
H2
L2
H3
L3
H4
Δδ / ppm
Residues
C
H1 helix
L1 loop
H2 helix
L2
H3 helix
L3 loop
H4 helix
disordered tail
MACPLEKALDVMVSTFHKYSGKEGDKFKLNKSELKELLTRELPSFLGKRTDEAAFQKLMSNLDSNRDNEVDFQEYCVFLSCIAMMCNEFFEGFPDKQPRKK
K31
L9
G47

Table S1. Experimentally (NMR spectroscopy) detected long range NOE constraints and MD simulations regarding fulfillment of these constraints. Percent of snapshots in compliance with the long distance NOE results.

long distance NOE	percent of snapshots with given distance < 6Å	
	Model A	Model B
W23-HN … D21-HB*	93.3	93.5
K24-HN … D21-HB*	94.8	96.8
L25-HN … D21-HB*	84.0	84.4
L25-HN … E28-HB*	96.6	97.5
L26-HN … E28-HB*	97.4	99.6
P36-HD* … Q38-HN	98.9	89.6
P36-HD* … A39-HN	36.9	100.0
A39-HN … L43-HD*	100.0	80.8
A39-HB* … D41-HN	59.9	95.0
A39-HB* … L43-HD*	99.3	24.1
M40-HN … L43-HD*	88.1	97.7
D41-HN …L43-HB*	54.5	34.1
D41-HN …L43-HD*	88.1	97.7
D42-HN … M40-HB*	100.0	80.7
M44-HN … D42-HN	100.0	100.0
L45-HN … L43-HD*	99.9	85.0

Table S2: The clamp model in other protein systems with an IDP and the partner molecule.

IDP	Partner	Reference in SI
Ste5p	Fus3p	S1
Oct-1 transcription factor	Ig-K promoter	S2
NLS	α-Importin	S3, S4
Cellulase E	Cellulose	S5
Myosin VI	Actin filament	S6
UPF2	UPF1	S7

References

[S1] R. P. Bhattacharyya, A. Reményi, M. C. Good, C. J. Bashor, A. M. Falick, W. A. Lim, *Science* **2006**, *311*, 822-826.

[S2] H. C. van Leeuwen, M. J. Strating, M. Rensen, W. de Laat, P. C. van der Vliet, *EMBO J.* **1997**, *16*, 2043–2053.

[S3] M. R. Fontes, T. Teh, B. Kobe, *J. Mol. Biol.* **2000**, *297*, 1183-1194.;

[S4] M. R. Fontes, T. Teh, G. Toth, A. John, I. Pavo, D. A. Jans, B. Kobe, *Biochem. J.* **2003**, *375*, 339-349.

[S5] I. von Ossowski, J. T. Eaton, M. Czjzek, S. J. Perkins, T. P. Frandsen, M. Schülein, P. Panine, B. Henrissat, V. Receveur-Bréchot, *Biophys. J.* **2005**, *88*, 2823–2832.

[S6] R. S. Rock, B. Ramamurthy, A. R. Dunn, S. Beccafico,B. R. Rami, C. Morris, B. J. Spink, C. Franzini-Armstrong, J. A. Spudich, L. A. Sweeney, *Mol. Cell* **2005**, *17*, 603–609.

[S7] M. Clerici, A. Mourão, I. Gutsche, N. H. Gehring, M. W. Hentze, A. Kulozik, J. Kadlec, M. Sattler, S. Cusack, *EMBO J.* **2009**, *28*, 2293–2306.
